# Microbial community succession during crude oil-degrading bacterial enrichment cultivation and construction of a degrading consortium

**DOI:** 10.3389/fmicb.2022.1044448

**Published:** 2022-11-04

**Authors:** Tianfei Yu, Xiaodong Liu, Jiamin Ai, Jiamin Wang, Yidan Guo, Xinhui Liu, Xiaolong He, Zhenshan Deng, Yingying Jiang

**Affiliations:** College of Life Sciences, Yan’an University, Yan’an, China

**Keywords:** crude oil, biodegradation, consortium, *Rhodococcus*, *Pseudomonas*

## Abstract

Microbial community succession during the enrichment of crude-oil-degrading bacteria was analyzed using Illumina high-throughput sequencing to guide bacterial isolation and construction of a bacterial consortium. Community change occurred in 6 days; the most abundant phylum changed from Proteobacteria to Actinobacteria; the most abundant genera were *Dietzia* and *unspecified_Idiomarinaceae*. Two crude oil-degrading strains, *Rhodococcus* sp. OS62-1 and *Dietzia* sp. OS33, and one weak-crude-oil-degrading strain, *Pseudomonas* sp. P35, were isolated. A consortium comprising *Rhodococcus* sp. OS62-1 and *Pseudomonas* sp. P35 showed the highest crude-oil-degrading efficiency, reaching 85.72 ± 3.21% within 7 days, over a wide pH range (5–11) and salinity (0–80 g·L^−1^). Consumption of saturated hydrocarbons, aromatic hydrocarbons, and resins was greater by the consortium than by a single strain, as was degradation of short-chain-alkanes (C_13_–C_17_) according to gas-chromatography. The bacterial consortium provides technical support for bioremediation of crude oil pollution.

## Introduction

Crude oil is one of the essential compounds in both the energy and chemical industries (Li et al., 2016). However, crude oil leakage caused by natural or human factors results in serious environmental pollution and sustainability problems worldwide (Gu and Wang, 2015; Muthukumar et al., 2022). Most of the components in crude oil can be stored and bioaccumulated in animals and humans, causing serious health issues (Elumalai et al., 2021). Degradation of crude oil pollutants is slow under natural conditions (Varjani and Upasani, 2019). There are numerous methods to repair crude oil pollution, including physicochemical and biological methods (Prendergast and Gschwend, 2014). However, physicochemical methods possess several side effects. Microbial remediation has attracted considerable attention because it is environmentally friendly, economical, and efficient (Kuyukina et al., 2013; Govarthanan et al., 2020).

Because of the complexity of crude oil composition, the biodegradation efficiency by a single microbe is extremely low (Li et al., 2016). Owing to the synergistic effect between strains, consortia possess a wide range of substrate spectra, higher robustness, and better adaptation to complex environments (Varjani and Upasani, 2019). The bacterial consortium constructed using surfactant producer *Acinetobacter* sp. Y2 and crude oil-degrading *Scedosporium* sp. ZYY possessed higher biodegradation rate and lower surface tension than the single strain (Atakpa et al., 2022). The bacterial consortium constructed using *Dietzia* sp. CN-3 and *Acinetobacter* sp. HC8-3S achieved higher crude oil degradation efficiency and better pH and NaCl tolerance than a single strain (Chen et al., 2020). Therefore, constructing a consortium is essential for microbial remediation of crude oil pollution (Balba et al., 1998; Suja et al., 2014).

To date, due to the lack of relevant theoretical guidance, most of the constructed consortiums are compounded between several crude oil-degrading bacteria, challenging to adapt to the real polluted environment. In fact, in crude oil-contaminated environments, only a tiny proportion of microbes can degrade crude oil, most of which are non or weak oil-degrading microbes (Aislabie et al., 2006). Enrichment culture procedures are usually required before isolating crude oil-degrading bacteria (Phulpoto et al., 2021). Under the stimulation of nutrients and crude oil, microbial communities will undergo the corresponding succession, and crude oil-degrading bacteria will be further enriched. Therefore, exploring the community succession of crude oil degrading bacteria enrichment culture and revealing the microbial community structure under enrichment culture will help to provide a reference for the construction of consortium in the selection of strains. With the development of high-throughput sequencing technology, the microbial diversity in crude oil-contaminated soil (Bidja Abena et al., 2020; Wang et al., 2020; Huang et al., 2021), water bodies (Harayama et al., 2004), and sediments (Chen et al., 2017) has been continuously described. However, the changes in the microbial community during the process of enrichment cultivation is poorly understood.

In the present study, Illumina high-throughput sequencing technology was used to analyze microbial community succession during the enrichment process to guide the isolation of microbes from the enrichment culture and construction of a crude-oil-degradation consortium. Then, the degradation effects of the consortium and single strain were evaluated by first-order kinetics, gas chromatography analysis (GC), and chromatographic separation. Finally, the tolerance of the consortium and single strain to NaCl and pH was also tested. This study provides a theoretical basis for utilizing microorganisms in the remediation of crude oil pollution.

## Materials and methods

### Chemicals and media

All chemicals used in the study were of analytical or high-purity grade. Crude oil (density: 0.742 g·cm^−3^) and contaminated soil (crude oil content: 1328.451 mg·kg^−1^) were obtained from the YANCHANG Oilfield in northern Shaanxi, China. The crude oil-contaminated soil was collected in a sterilized polyethylene bottle, immediately transported to the laboratory, and stored at 4°C until further use.

Two different culture media were used in this study. The enrichment medium (ERM) used for the enrichment cultivation, microbial isolation, and biodegradation test in this study contained (NH_4_)_2_SO_4_ (1 g·L^−1^), NaNO_3_ (2 g·L^−1^), KH_2_PO_4_ (5 g·L^−1^), MgSO_4_·7H_2_O (0.3 g·L^−1^), NaCl (5 g·L^−1^), microelements (1 ml, Supplementary Table S1), and crude oil (10 g·L^−1^) at pH 8.5. Peptone yeast glucose (PYG) medium was used for the strain proliferation, which composition was as follows: peptone (5 g·L^−1^), yeast extract (0.5 g·L^−1^), glucose (5 g·L^−1^), beef extract (3 g·L^−1^), MgSO_4_·7H_2_O (1.5 g·L^−1^) and NaCl (10 g·L^−1^) at pH 7.5. All media were sterilized at 121°C for 15 min before use.

### Methods

#### Enrichment cultivation

Crude oil-contaminated soil was treated to remove impurities, such as small stones, roots, and leaves, and was subsequently mixed completely. As a control, 5 g of soil sample was collected and stored at −80°C until further use. For enrichment cultivation, a 10 g soil sample was added to 1 l ERM and cultured at 28°C and 180 rpm. Every 3 days, 40 ml of the enrichment culture was collected, 30 ml of the culture was centrifuged at 12,000 rpm for 20 min at 4°C to obtain the pellet, and 10 ml was stored at 4°C for bacterial isolation. The pellets were stored at −80°C until the end of enrichment cultivation (15 days). Three replicate experiments were performed. The frozen soil sample and collected enrichment culture pellets were used for DNA extraction, and Illumina high-throughput sequencing that was performed at Wekemo Tech Co., Ltd. (Shenzhen, China).

#### Illumina high-throughput sequencing

The microbial communities in the crude oil-contaminated soil and pellets collected from the enrichment culture were sequenced using the Illumina Novaseq platform (Illumina, San Diego, CA, United States). Soil, ERC1, ERC2, ERC3, ERC4, and ERC5 represent soil samples and enrichment cultures on days 3, 6, 9, 12, and 15, respectively. DNA from all samples was extracted using the E.Z.N.A.^®^ Soil DNA Kit (Omega Bio-Tek, Norcross, GA, United States) according to the manufacturer’s protocol. The V3-V4 hypervariable regions of the 16S rRNA gene were amplified using primers 341F (5′-CCTAYGGGRBGCASCAG-3′) and 806R (5′-GGACTACHVGGGTWTCTAAT-3′; Niem et al., 2020). The DNA libraries were constructed using FastPfu Polymerase for PCR to amplify DNA, which was then purified using the AxyPrep DNA Gel Extraction Kit (Axygen Biosciences, Union City, CA, United States) and quantified using QuantiFluor™-ST (Promega, United States) according to the manufacturer’s protocol.

The raw reads were analyzed using the QIIME2 dada 2.0 plugin to obtain the feature table of amplicon sequence variants (ASVs), exact sequence variants, or sub-operational taxonomic units (sub-OTUs; Callahan et al., 2016). Different features contained different sequences at the single-nucleotide level. To minimize the effects of sequencing depth on data analysis, the number of reads in each sample was rarefied to 38,834. The generated ASVs were aligned to the GREENGENES 13_8 database classifier with 99% similarity using the QIIME2 feature-classifier plugin to generate a taxonomic table (Bokulich et al., 2018). Contaminated mitochondrial and chloroplast sequences were filtered and removed using the QIIME2 feature-table plugin.

The 16S rRNA Illumina libraries were deposited in the NCBI small read archive (SRA) dataset under the BioProject accession numbers PRJNA776725 and SRA accession numbers SRR16702634-SRR16702651.

#### Isolation and identification of bacteria

The 10 ml enrichment cultures were homogenized, and 1 ml of each enrichment culture was taken out and mixed thoroughly. The mixed bacterial suspension was used for bacterial isolation using the dilution-plating method on the ERM and PYG media.

Cell morphology was examined using scanning electron microscopy (JSM-7610f, JEOL, Japan) and Gram staining. For 16S rRNA gene sequencing and phylogenetic analysis, 16S rRNA gene sequences were obtained as previously described (Deng et al., 2020) and the GenBank accession numbers of strains OS62-1, OS33, and P35 were MZ149267, MZ149266, and ON115028, respectively. The 16S rRNA gene sequence of the strain was compared with those available from Ezbiocloud database (Chun et al., 2007). The phylogenetic tree was analyzed by MEGA 5.1 using the Neighbour-Joining (N-J) method (Hollingsworth and Ennos, 2004).

#### Crude-oil-degradation test

To evaluate the capability of the isolated microbial strains to biodegrade crude oil, the purified strains were first inoculated into fresh PYG medium and cultured at 28°C and 160 rpm for 2 days. After centrifugation at 8,000 rpm for 5 min, the cells were collected and washed 2–3 times using sterile water. The concentration of the bacterial solution was adjusted to OD_600_ = 1.00 for standby. The proportion of bacteria in the consortium was 1:1 throughout the entire experimental process. Then, the microbial (0.1%, v/v) was added to ERM and cultured at 160 rpm for 7 days. Finally, the residual crude oil was extracted with an equal volume of C_2_Cl_4_, dried in anhydrous sodium sulfate, and concentrated *via* vacuum rotary evaporation. The residual crude oil concentration was determined using an infrared spectrometric oil detector (OIL-480, China Invent, China). The crude-oil-biodegradation rate formula is as follows:


$$\eta =\frac{C_{0}-C_{t}}{C_{0}}\times 100\%$$

where *η* is the biodegradation efficiency of crude oil (%), *C_0_* is the initial crude oil concentration in the medium (mg·L^−1^), and *C_t_* is the crude oil concentration in the medium at time *t* (mg·L^−1^).

#### Kinetics of crude-oil-degradation in microcosm

To further evaluate the biodegradation characteristics of crude oil by the consortium and the single strain, the concentration of residual crude oil and strain growth were measured at an interval of 1 day. Strain growth was evaluated using the plate counting method on PYG agar plates. Crude-oil-degradation was determined using a first-order kinetic model (Kachienga and Momba, 2017). The formula used is as follows:


$$InCt=InC_{0}-kt$$

$$t_{1/2}=In2/k$$

Specifically, *t* is the degradation time (days), *Ct* is the crude oil concentration at time (*t*), *C_0_* is the initial crude oil concentration, *k* is the degradation rate constant (day^−1^), *t_1/2_* is the half-life of crude-oil-degradation (days).

#### GC analysis

The components of residual crude oil after biodegradation by single strains and the bacterial consortium were measured using GC (7820A, Agilent Technologies, United States) with a capillary column (HP-5 model, 30 m × 0.25 mm × 0.25 μm). The operating conditions were as follows: helium was used as the carrier gas at a flow rate of 2 ml·min^−1^; the column temperature was 60°C for 1 min, with a ramp to 290°C t a rate of 8°C min^−1^, raised to 320°C at a rate of 30°C·min^−1^, which was maintained for 7 min. Petroleum hydrocarbon mixed standards (1,000 μg·mL^−1^) were used as internal standards to calibrate GC measurements (Liu et al., 2009).

#### Degradation of different crude oil components

The chromatographic separation of saturated, aromatic, resin, and asphaltene fractions in residual crude oil after biodegradation followed the method described by Márquez et al. (1999). Briefly, the residual crude oil after biodegradation was dissolved in 30 ml n-hexane, asphaltene was filtered by degreasing cotton and dissolved in CHCl_3_. The filtrate was then concentrated to 2 ml and added to a glass column containing neutral alumina (200–300 mesh, Kermel, China). The saturated fraction was extracted with 30 ml n-hexane, yielding a colorless fraction. The aromatic fraction was extracted using a 2:1 (v/v) CH_2_Cl_2_ / C_6_H_14_ mixture (20 ml), yielding a yellowish fraction. The resin fraction was first separated with 10 ml ethanol and then extracted with 10 ml chloroform. The solvents in the fractions were evaporated and weighed.

#### Effect of culture conditions on crude-oil-biodegradation

Crude-oil-biodegradation of the potent isolate was carried out in the presence of different pH and salinity levels using a one-variable-at-a-time approach, keeping other parameters constant. Different pH (3.0–11.0) and salinity (0–70 g·L^−1^) values were prepared in the ERM. The bacterial consortium and single strain were inoculated into each medium (0.1%, v/v) and then cultured at 28°C and 180 rpm for 7 days to determine the concentration of remaining crude oil.

### Statistical analysis

All experiments were performed in triplicate, and the data are expressed as mean ± standard deviation (SD). ORIGIN 2020, SPSS 22.0, and R 4.1.3 software were used for data analysis and statistics, with *p <* 0.05, indicating a significant statistical difference.

## Results and discussion

### Microbial diversity during enrichment cultivation

Illumina high-throughput sequencing generated 1,304,557 high-quality reads from 6,244 features at single-nucleotide resolution. After rarefication to 38,834 reads for each sample, 5,825 features were generated from all 18 samples.

The crude oil-containing soil samples showed significantly higher observed_OTUs index and Shannon index than the enrichment samples (Supplementary Table S2). The observed_OTUs decreased sharply from 1,548 ± 185 in the soil samples to 153 ± 2 in the enrichment samples on day 3 (ERC1). However, when enrichment was continued to days 6, 9, and 12, the observed_OTUs index increased to 294 ± 118, 217 ± 70, and 630 ± 232, respectively. Then, on day 15, the observed_OTUs index decreased to 179.866 ± 57.960. Similarly, the Shannon index for the soil samples was higher than that of the enrichment cultures and was reduced from 7.902 ± 0.551 in the soil samples to 2.788 ± 0.169 in ERC1. The lowest Shannon index was observed on day 9 (1.298 ± 0.306). The other alpha diversity indices, including the Chao1, Faith_PD, and Simpson indices, showed similar trends (Supplementary Table S2). Thus, as described in previous studies (Jin and Kim, 2017; Mu et al., 2018), the microbial alpha diversity in the enrichment culture samples was considerably lower than that in the soil samples.

### Microbial community structure

**Figure 1:**
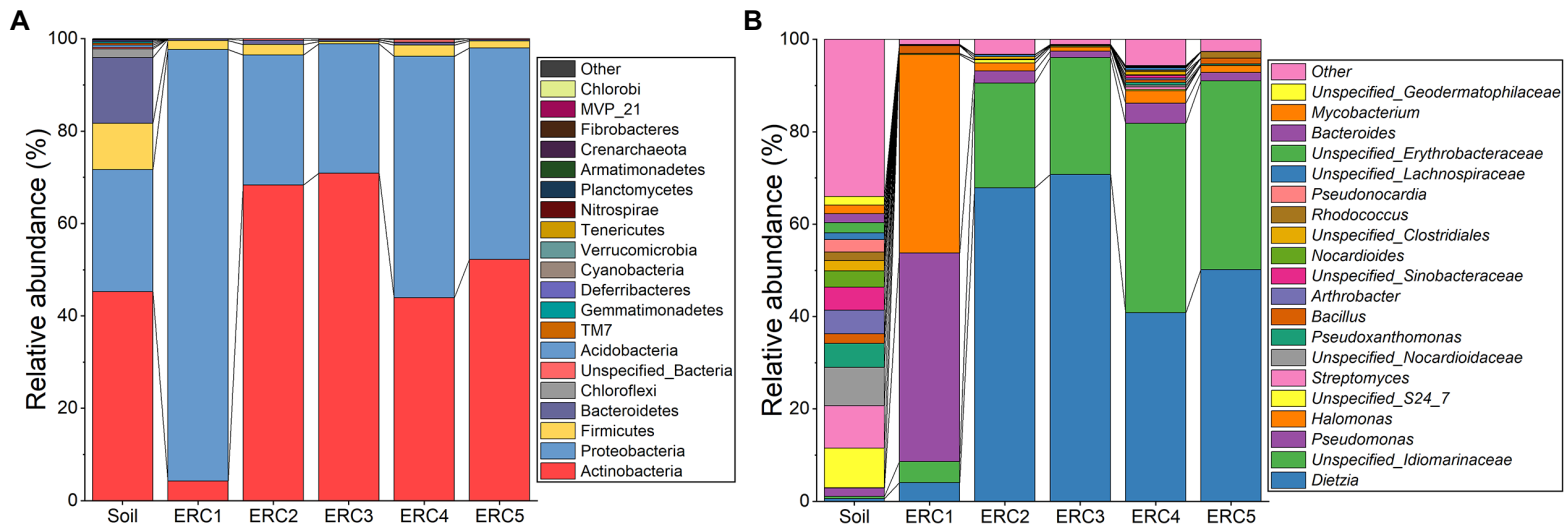
Microbial community composition during enrichment culture at **(A)** phylum and **(B)** genus levels (relative abundance top 20).

We examined bacterial taxa and their relative abundances in the soil and enrichment culture samples at different time points. In soil, the predominant phyla were Actinobacteria (45.20 ± 7.25%), Proteobacteria (26.48 ± 4.46%), Firmicutes (10.00 ± 1.80%), and Bacteroidetes (14.24 ± 11.02%; Figure 1A). Unlike the microbial community composition described in the investigation of historic oilfield-contaminated soil in the Loess Plateau (Gao et al., 2019), the dominant phylum in this study was Actinobacteria, rather than Proteobacteria, possibly owing to differences in contamination levels. In ERC1, Proteobacteria predominated (93.39 ± 1.29%), followed by Actinobacteria (4.28 ± 1.32%), and Firmicutes (1.95 ± 0.58%). The relative abundances of the other phyla in ERC1 were below 1%. In ERC2-5, Actinobacteria and Proteobacteria were the predominant phyla (58.86 ± 19.72% and 38.52 ± 19.40%, respectively). Compared to ERC1, the relative abundance of Actinobacteria was significantly higher in ERC2-5 (Student’s *t*-test, *p* = 0.003, 0.011, 0.037, 0.048 for ERC2-5 compared to ERC1, respectively), whereas that of Proteobacteria was significantly lower (Student’s *t*-test, *p* = 0.001, 0.012, 0.041, 0.046 for ERC2-5 compared to ERC1, respectively). Despite fluctuations in relative abundance, Actinobacteria and Proteobacteria were the predominant phyla in ERC2-5 (Figure 1A).

The top 20 identified genera are shown in Figure 1B. A noticeable difference was observed in the distribution of genera among the soil, ERC1, and ERC2-5 samples. The relative abundance of the top 20 genera in the soil samples displayed a more uniform distribution than those in the ERC samples. In ERC1, *Pseudomonas* and *Halomonas* showed high relative abundances (45.13 ± 10.90% and 42.88 ± 11.78%, respectively), followed by *Dietzia* and an *unspecified_Idiomarinaceae* genus (4.07 ± 1.27% and 4.58 ± 0.90%, respectively; Figure 1B). In ERC2, the relative abundances of *Dietzia* and *unspecified_Idiomarinaceae* were increased dramatically, to 67.91 ± 7.92% and 22.63 ± 2.34%, respectively. ERC3-5 showed a bacterial composition similar to that of ERC2.

The composition of microbial communities reached a relatively steady state on day 6 of enrichment cultivation, indicating that a duration of 1 week was sufficient for enrichment cultivation under these conditions. *Dietzia* sp. have been reported to be marker microorganisms of crude oil pollution, and many *Dietzia* sp. have an excellent ability to degrade crude oil (Wang et al., 2014; Huang et al., 2021; Venil et al., 2021). The high abundance of the genera *Halomonas* and *Pseudomonas* in ERC1 implied they played important roles in the initial stages of crude-oil-degradation, whereas the abundance of *Dietzia* and *unspecified_Idiomarinaceae* suggested that their roles were important in the later stages of crude-oil-degradation. Unfortunately, no strains belonging to *unspecified_Idiomarinaceae* and *Halomonas* were isolated in this study, and their roles could not be investigated.

### Biodegradation of crude oil by defined consortium

Three kinds of bacteria, OS62-1, OS33, and P35, were obtained; OS62-1 and OS33 were isolated using ERM medium and P35 was isolated using PYG medium. After 3 days of incubation on PYG agar plates, strain OS62-1 formed circular, smooth, pink colonies (0.5–1.0 mm). The strain OS62-1 was Gram-positive. Scanning electron microscopy revealed that it was a coccus (0.5–1.0 μm) bacterium. The strain OS33 was a Gram-positive bacilli bacterium (2.0 × 0.5 μm), forming wet, orange and opaque colonies (0.5–1.0 mm). Strain P35 formed wet, light yellow, opaque, and shiny colonies (1.0–2.0 mm), which were Gram-negative bacilli (3.0 × 1.0 μm; Supplementary Figure S1).

**Figure 2:**
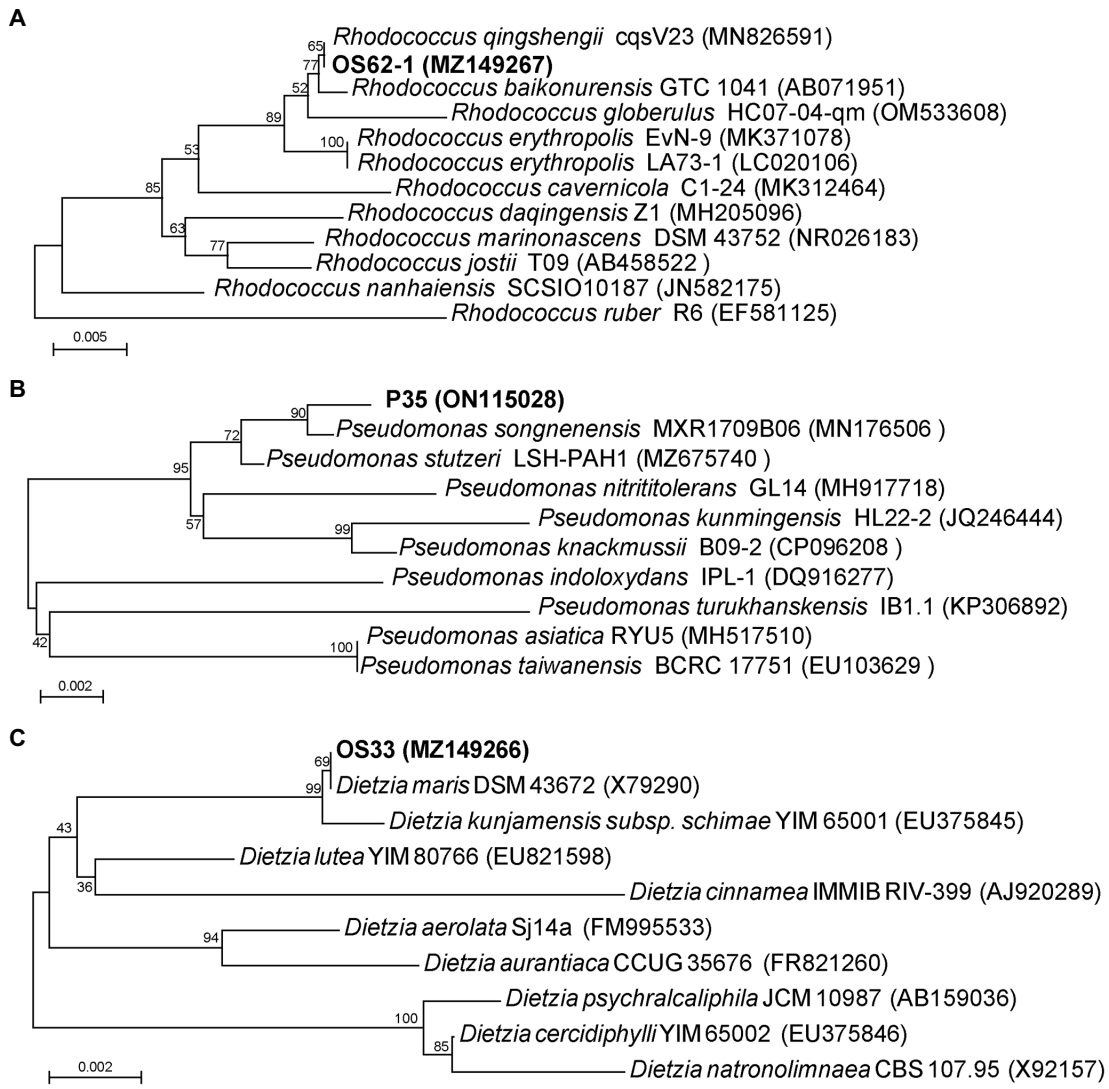
Phylogenetic tree based on 16S rRNA sequences of OS62-1 **(A)**, P35 **(B)**, OS33 **(C)** and related strains. Bar = 0.01 nucleotide substitution per locus.

The 16S rRNA gene sequence comparisons indicated that strain OS62-1 showed a high similarity (100%) to the previously isolated bacterium *Rhodococcus qingshengii* cqsV23. The strain OS33 possessed 100% sequence similarity with the strain *Dietzia maris* DSM 43672. The strain P35 possessed 99.51% sequence similarity with the strain *Pseudomonas songnenensis* MXR1709B06. Phylogenetic trees based on the 16S rRNA sequences of strains OS62-1, OS33, and P35 were constructed using neighbor-joining (Figures 2A–C). Based on the phylogenetic analysis results, strains OS62-1, OS33, and P35 were tentatively suggested to be members of the genera *Rhodococcus*, *Dietzia* and *Pseudomonas*, respectively.

**Figure 3:**
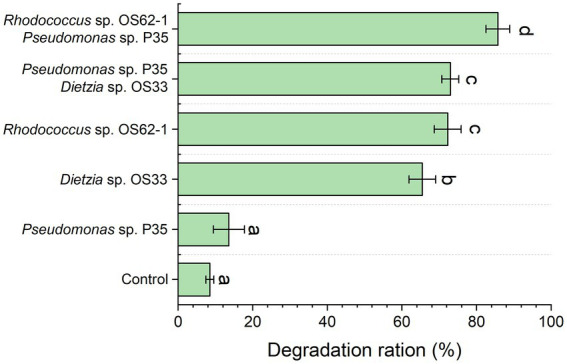
The crude oil degradation efficiency of the isolated strains and the constructed consortium was tested for 7 days. Groups sharing different letters (a, b, c) indicate significant differences between two treatments by one-way ANOVA test (*p* < 0.05).

The propagation speed and morphology of *Rhodococcus* sp., OS62-1, and *Dietzia* sp. OS33 were good during the biodegradation of crude oil. As shown in Figure 3, the biodegradation rates of *Rhodococcus* sp. OS62-1, *Dietzia* sp. OS33, and *Pseudomonas* sp. P35 over 7 days were 72.28 ± 3.60%, 65.46 ± 3.57%, and 13.61 ± 4.12%, respectively. It has been extensively reported that *Rhodococcus* is one of the groups with most potential for biodegradation and it can degrade crude oil, especially aromatic compounds (Li et al., 2020). In this study, the control group also exhibited an 8.51 ± 1.06% degradation rate, which may be due to the loss of crude oil in the extraction process. Although many *Pseudomonas* sp. were proved to be good crude-oil-degrading bacteria, the crude-oil-degradation efficiency of *Pseudomonas* sp. P35 was only slightly higher than that in the control group, suggested that the *Pseudomonas* sp. P35 can be considered a very weak-oil-degrading bacterium.

To further study the role of the weak oil-degrading bacterium *Pseudomonas* sp. P35, we combined it with *Rhodococcus* sp. OS62-1 and *Dietzia* sp. OS33 in a 1:1 ratio to construct a bacterial consortium and tested the crude-oil-degradation efficiency. *Pseudomonas* sp. P35 formed good symbiotic relationships with *Rhodococcus* sp. OS62-1 and *Dietzia* sp. OS33 and the crude-oil-degradation efficiency over 7 days increased to 85.72 ± 3.21% and 72.97 ± 2.30%, respectively (Figure 3). Although some studies have shown that *Pseudomonas* can promote the degradation ability of some petroleum degrading bacteria (Isaac et al., 2015; Hu et al., 2020; Feng et al., 2021), our study showed that *Pseudomonas* had different promoting effects on different petroleum degrading bacteria. The *Pseudomonas* sp. P35 had a stronger ability to promote the crude oil degradation of *Rhodococcus* sp. OS62-1 than *Dietzia* sp. OS33. Therefore, we selected *Rhodococcus* sp. OS62-1 and *Pseudomonas* sp. P35 for further study.

### Kinetics of crude-oil-degradation

**Figure 4:**
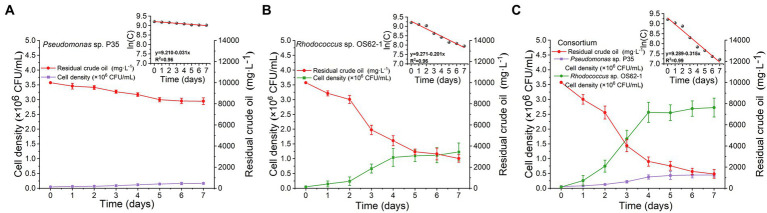
Time course of strain cell density and crude oil biodegradation by **(A)** *Pseudomonas* sp. P35, **(B)** *Rhodococcus* sp. OS62-1 and **(C)** Consortium in 10,000 mg·L^−1^ crude oil amended ERM. The values represent the average of two independent experiments with three replicates each.

Microcosms for crude-oil-biodegradation were prepared by inoculating *Rhodococcus* sp. OS62-1, *Pseudomonas* sp. P35, and the consortium into 100 ml of ERM amended with 10,000 mg·L^−1^ crude oil. The biodegradation rate of crude oil in the ERM inoculated with *Pseudomonas* sp. P35 was extremely weak (Figure 4A); its rate constant (*k*) was only 0.031 d^−1^ (*R^2^* = 0.96), and its half-life (*t_1/2_*) was 22.36 d (Table 1). On the second day, the crude oil inoculated with *Rhodococcus* sp. OS62-1 dispersed in the ERM as tiny particles, the turbidity of the medium increased with obvious emulsification, and the degradation rate of crude oil accelerated (Figure 4B). The rate constant (*k*) was 0.201 d^−1^ (*R^2^* = 0.96), and the half-life (*t_1/2_*) was 3.45 d (Table 1). Unfortunately, the biosurfactants produced by *Rhodococcus* sp. OS62-1 were not successfully separated, suggested that the surfactants might attached to the cells tightly. Compared to the single strain, the crude oil degradation rate constant (*k*) of the consortium increased to 0.315 d^−1^ (*R^2^* = 0.99), and the half-life (*t_1/2_*) was shortened to 2.20 d (Table 1). The biomass of *Rhodococcus* sp. OS62-1 and *Pseudomonas* sp. P35 also increased significantly in the consortium than in the single strain tests (Figure 4C), suggested that the two strains can assist each other in crude oil degradation.

**Table 1:** First-order kinetics equations and the corresponding half-life times of crude oil biodegradation by *Rhodococcus* sp. OS62-1, *Pseudomonas* sp. P35, and Consortium at 28°C in 7 days.

Treatment	Exponential equation	*R^2^*	*t_1/2_*
*Pseudomonas* sp. P35	*C_t_* = 9.210e^−0.031t^	0.96*	22.36
*Rhodococcus* sp. OS62-1	*C_t_* = 9.271e^−0.201t^	0.96*	3.45
Consortium	*C_t_* = 9.289e^−0.315t^	0.99*	2.20

*^#^C_t_*: the residual crude oil concentration at time *t* (mg·L^−1^); *t_1/2_*: time to reach 50% biodegradation of crude oil (day); *significant at 0.05 level.

It is important to evaluate the biodegradation kinetics of crude oil to develop effectively and predicted bioremediation models (Chettri and Singh, 2019). In this study, the crude oil concentration decreased with incubation time, and the data were in good agreement with the first-order kinetic equation (*R^2^* > 0.95). There are several reports on crude-oil-biodegradation by either a single strain or a consortium fitted to first-order reaction kinetics at low crude oil concentrations (Chen et al., 2008; Gu et al., 2016). Moreover, the consortium and *Rhodococcus* sp. OS62-1 degraded crude oil at a relatively high concentration for a short period of 7 days. However, when incubated for longer than 4 days, the degradation efficiency of crude oil began to slow owing to nutrient deficiency (Singh et al., 2014) or accumulation of toxic substances (Chettri et al., 2016).

### GC analysis

**Figure 5:**
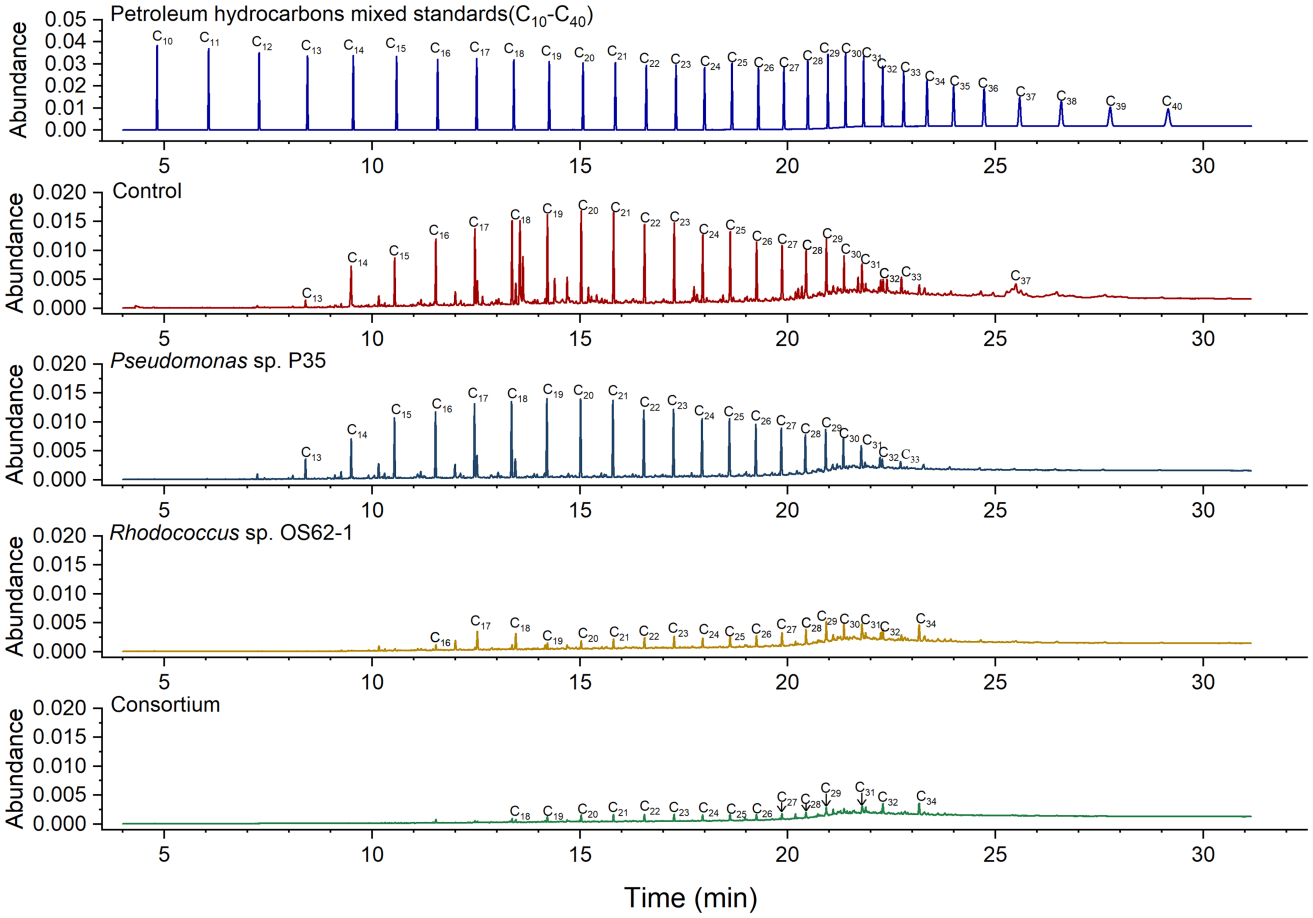
Gas chromatogram (GC) of biodegradation efficiency of saturated hydrocarbons in crude oil by single strain and consortium treatment in 7 days.

The biodegradation ratios of n-alkanes (C_13_–C_37_) in crude oil were calculated by semi-quantitative analysis of the GC data for residual and fresh crude oil. According to previous studies (Li et al., 2013), the carbon atom number ranges of short-chain alkanes, long-chain alkanes, and heavy long-chain alkanes are considered to be C_10_–C_17_, C_18_–C_30_ and C_31_–C_38_, respectively. The concentrations of only a few long-chain alkanes (C_17_–C_21_) were slightly decreased by *Pseudomonas* sp. P35 (Figure 5), providing further evidence for its weak oil-degrading bacteria status. *Rhodococcus* sp. OS62-1 can use C_13_–C_33_ n-alkanes in crude oil as carbon sources, similar to *R. erythropolis* M-25 and *R. zopfii* P2-29 (Pi et al., 2017). The biodegradation ratio of the total crude oil by the consortium was 85.84%, which was 10.76% more than that of the *Rhodococcus* sp. OS62-1 (75.08%). More specifically, 94.61% of short-chain alkanes, 77.57% of long-chain alkanes, and 36.09% of heavy long-chain alkanes were degraded by the consortium. Although *Rhodococcus* sp. OS62-1 performed well in the degradation of short-chain alkanes, the consortium seemed to have a higher degradation efficiency especially for C_13_-C_17_ n-alkanes, which almost completely disappeared, illustrating the powerful performance of the consortium.

### Measurement of crude oil components

**Figure 6:**
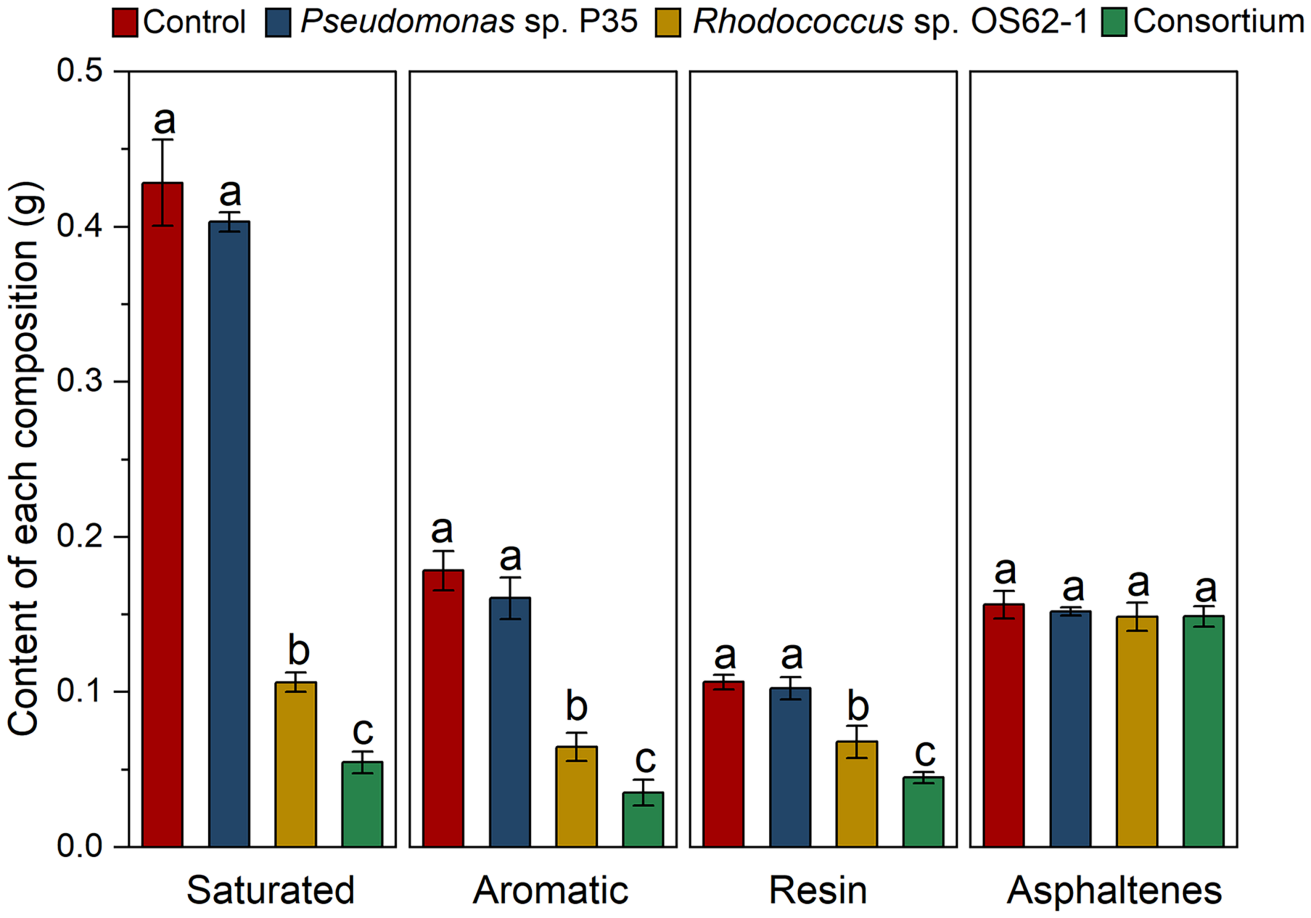
Changes of contents of different components in crude oil under different treatments in 7 days. Groups sharing different letters (a, b, c) indicate significant differences between two treatments by one-way ANOVA test (*p* < 0.05).

Biodegradation of different components of crude oil by single strain and the consortium was analyzed using chromatographic separation. The contents of the diverse components are displayed in Figure 6. The biodegradation efficiency of the consortium was significantly higher (*p* < 0.05) than that of the single strain for the different components. The contents of saturated alkanes decreased from 0.428 ± 0.028 g to 0.055 ± 0.028 g after treatment with the consortium, which was significantly higher (*p* < 0.05) by more than 48.11% compared to the *Rhodococcus* sp. OS62-1 (0.106 ± 0.062 g). A similar situation also occurred for aromatic hydrocarbons, the content of aromatic hydrocarbons in residual crude oil after treatment with the consortium was 0.035 ± 0.008 g, which was significantly lower (*p* < 0.05) than the decrease with *Rhodococcus* sp. OS62-1 (0.065 ± 0.009).

Owing to their complex structures, resins and asphaltenes in crude oil are extremely difficult to degrade (Ding et al., 2021). Excitingly, resin content also decreased significantly after treatment by *Rhodococcus* sp. OS62-1 and consortium. At present, there have been many reports on the application of microorganisms to solve the problem of resin deposition in crude oil transportation (Patel and Bhaskaran, 2018; Wang et al., 2022). The main applied bacteria are *Bacillus* sp., *Pseudomonas* sp., *Ochrobactrum* sp., but the reports on the degradation of resin by *Rhodococcus* sp. are limited. In this study, *Rhodococcus* sp. OS62-1 significantly decreased the resin content in crude oil from 0.107 ± 0.005 g to 0.068 ± 0.011 g (*p* < 0.05). Meanwhile, the consortium also performed better than *Rhodococcus* sp. OS62-1 in resin removal, indicating that it had great potential in resin removal. Compared with resin, the number of heteroatoms in asphaltene is higher, and its degradation is more difficult (Ding et al., 2021). The asphaltene content in this study did not change significantly, as described by (Chaineau et al., 1995), who found that asphaltenes were completely retained after saturation and aromatic hydrocarbons were completely degraded by microorganisms.

### Effect of culture conditions on crude-oil-biodegradation

It is necessary to understand the effects of physical and chemical factors (e.g., pH and salinity) on biodegradation because these factors significantly affect the biological activity and biodegradation efficiency of microorganisms (Khanpour-Alikelayeh et al., 2020). The pH tolerance range of *Rhodococcus* sp. OS62-1 and *Pseudomonas* sp. P35 was 5–11, and the salinity tolerance range of *Pseudomonas* sp. P35 was 0–80 g·L^−1^ which was wider than that of *Rhodococcus* sp. OS62-1 (0–50 g·L^−1^). Because the resistance of microorganisms to environmental salinity above 30 g·L^−1^ expands the opportunities for their use in bioremediation of oil-contaminated ground and water ecosystems (Iminova et al., 2022), the halotolerant characteristics of these two strains will lay the foundation for their practical applications.

**Figure 7:**
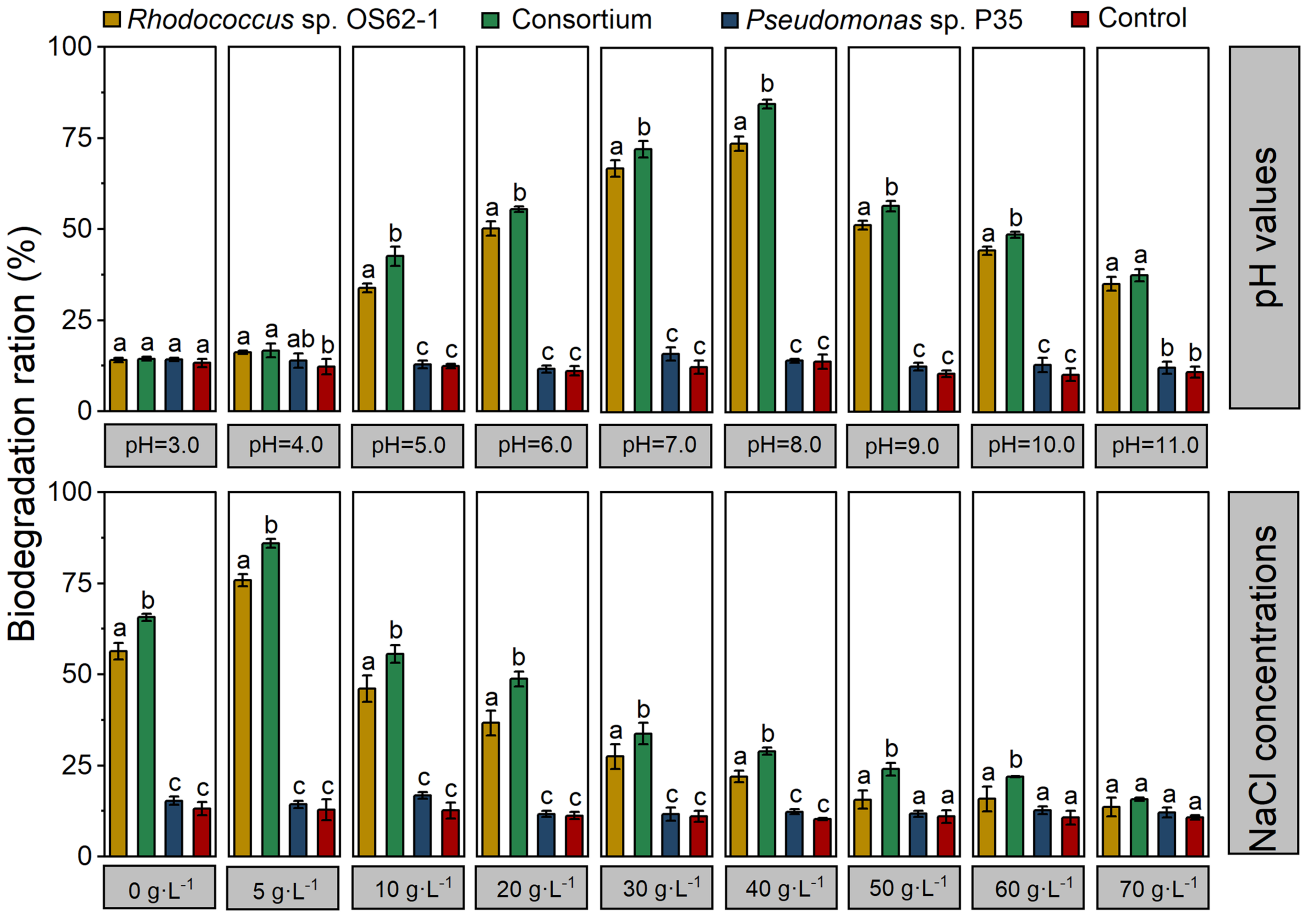
The degradation efficiency of crude oil by the single strain and consortium under different pH values and NaCl concentrations in 7 days. Groups sharing different letters (a, b, c) indicate significant differences between two treatments by one-way ANOVA test (*p* < 0.05).

Based on the adaptability of *Rhodococcus* sp. OS62-1, the effects of pH and salinity on crude-oil-biodegradation by a single strain and a consortium were studied. As shown in Figure 7, the crude-oil-biodegradation rates of *Rhodococcus* sp. OS62-1 were higher than 20% at NaCl concentrations ranging from 0–40 g·L^−1^; the optimum NaCl concentration was 5 g·L^−1^ (75.83 ± 1.73%). Because of the synergistic effects of *Rhodococcus* sp. OS62-1 and *Pseudomonas* sp. P35, the crude-oil-biodegradation efficiency of the consortium was significantly higher than that of the single strain in NaCl concentrations of 0–60 g·L^−1^ (*p* < 0.05). Like *Rhodococcus* sp. OS62-1, the optimal NaCl concentration for biodegradation by the consortium was also 5 g·L^−1^ (85.89 ± 1.21%). When the NaCl concentration was 70 g·L^−1^, no significant difference in crude oil content was detected between the treated and untreated flasks after 7 days of incubation.

To investigate effects of pH on crude-oil-degradation activity, experiments were carried out at different pH ranges (3–11). In the ranges of pH 5–10, the crude-oil-biodegradation efficiencies of the consortium were significantly higher than those of the single strain (*p* < 0.05), and varied from 42.54 ± 2.61% to 84.58 ± 1.21%. The biodegradation rate was higher than 55% within the pH range of 6–9, and the optimum pH was 8 (84.58 ± 1.21%). It is worth noting that the degradation efficiency of crude oil remained at 37.66% ± 1.67 in strong alkaline conditions (pH = 11).

Crude oil pollution is often accompanied by soil salinization (Huang et al., 2021), so the bacteria that are planned for use in the course of remediation must have salt and alkali resistance. According to previous studies, pH and NaCl concentrations are significant environmental factors affecting crude-oil-biodegradation (Kästner et al., 1998), and slightly saline (Zaki et al., 2015) and alkaline (Khanpour-Alikelayeh et al., 2020) conditions result in better hydrocarbon degradation performance. In this study, the constructed consortium showed the highest degradation efficiency at pH = 8.0 and NaCl concentration of 5 g·L^−1^; the pH tolerance range was 5–11 and the NaCl tolerance range was 0–60 g·L^−1^. Therefore, it can be concluded that this bacterial consortium can be used for real crude oil pollution of soil and water.

## Conclusion

The Illumina high-throughput sequencing results showed that the microbial community changes occurred within 6 days during the enrichment process and the *Dietzia* sp. OS33 and *Pseudomonas* sp. P35 possessed high abundance during the enrichment cultivation, while the *Rhodococcus* sp. OS62-1 possessed very low relative abundance throughout the enrichment process. However, the consortium composed of *Rhodococcus* sp. OS62-1 and *Pseudomonas* sp. P35 achieved the highest crude-oil-degradation efficiency. Based on the results of the first-order kinetic model, GC analysis, chromatographic separation, and NaCl and pH tolerance test, the constructed consortium has higher crude oil degradation efficiency and better environmental tolerance than a single strain. In summary, our results demonstrated the microbial changes during the enrichment process and constructed a consortium with high crude oil degradation efficiency and environmental tolerance.

## Data availability statement

The datasets presented in this study can be found in online repositories. The names of the repository/repositories and accession number(s) can be found in the article/Supplementary material.

## Author contributions

TY: experimentation and writing—original draft. YJ: conceptualization and methodology. JA: data curation and software. JW, XinL, and YG: experimentation. XH: methodology and resources. ZD: methodology and supervision. XiaoL: project administration, and writing—review and editing. All authors contributed to the article and approved the submitted version.

## Funding

This work was supported by Special Scientific Research Project of Shaanxi Education Department (no. 21JK0992); Special fund for science and technology of Yan’an City (no. 2019–27; no. 203010105); and Doctor Support Project of Yan’an University (no. YDBK2019-43).

## Conflict of interest

The authors declare that the research was conducted in the absence of any commercial or financial relationships that could be construed as a potential conflict of interest.

## Publisher’s note

All claims expressed in this article are solely those of the authors and do not necessarily represent those of their affiliated organizations, or those of the publisher, the editors and the reviewers. Any product that may be evaluated in this article, or claim that may be made by its manufacturer, is not guaranteed or endorsed by the publisher.
