# Supplementary material for: Microbial community succession during crude oil-degrading bacterial enrichment cultivation and construction of a degrading consortium
Source: Front Microbiol. 2022 Nov 4;13:1044448. doi: 10.3389/fmicb.2022.1044448 (PMC9672818; doi:10.3389/fmicb.2022.1044448)
Supplement: Supplementary file 1 [file Data_Sheet_1.docx]

**Supplementary Material**

**Captions**

Figure S1. Colony morphology, cell morphology and Gram staining results of the isolated strain on PYG medium in 3 days

Table S1 Microelements

Table S2 α-diversity index

Figure S1.


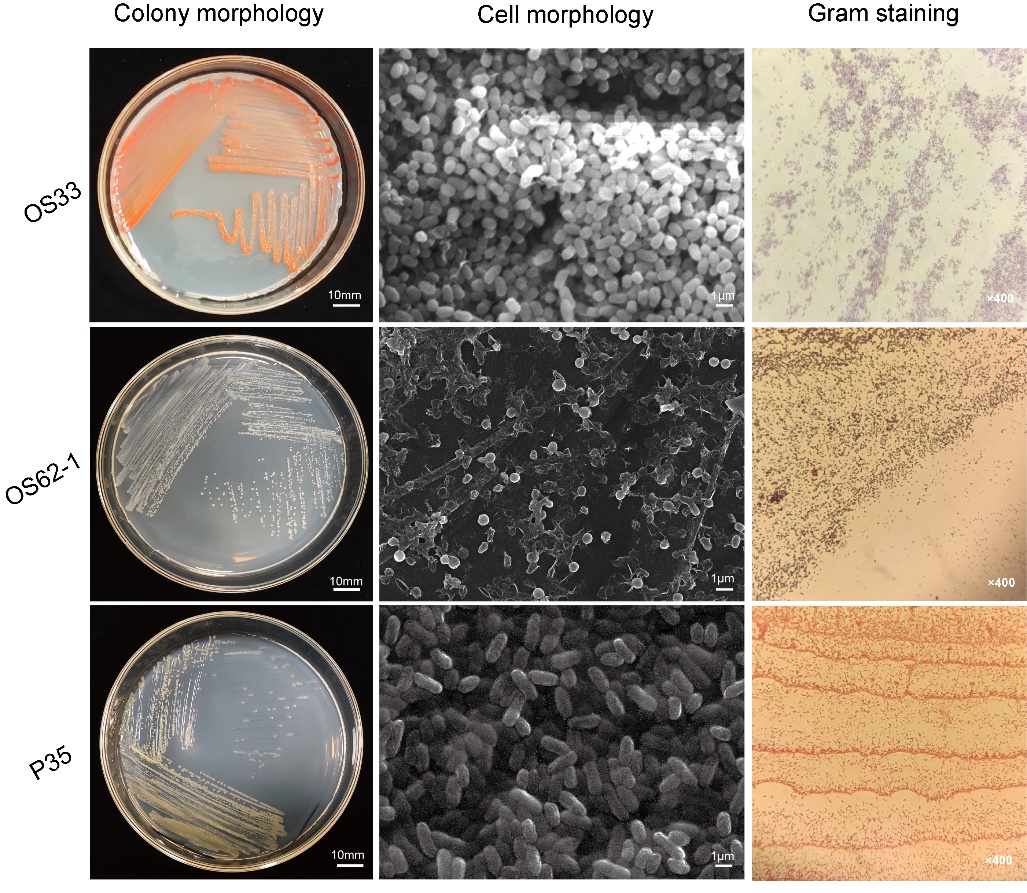


| Table S1 Microelements | |
| --- | --- |
| Chemicals | Content |
| ZnSO_4_·7H_2_O | 0.150 g |
| FeSO_4_·7H_2_O | 0.540 g |
| MnSO_4_·H_2_O | 0.020 g |
| CuSO_4_·5H_2_O | 0.035 g |
| CaCl_2_ | 0.015 g |
| H_3_BO_3_ | 0.005 g |
| Na_2_MoO_4_·2H_2_O | 0.010 g |
| ddH_2_O | 1000 mL |

^#^The solution was sterilized at 121°C for 15 min and stored in dark at 4 °C.

| Table S2 α-diversity index | | | | | |
| --- | --- | --- | --- | --- | --- |
| **Sample name** | **Chao1** | **Faith_pd** | **Observed_OTUs** | **Shannon** | **Simpson** |
| Soil | 1554.752 ± 182.871^a^ | 88.179 ± 1.668^a^ | 1548 ± 185^a^ | 7.902 ± 0.551^a^ | 0.983 ± 0.010^a^ |
| ERC1 | 153.108 ± 1.885^c^ | 19.195 ± 1.701^c^ | 153 ± 2^c^ | 2.788 ± 0.169^b^ | 0.786 ± 0.063^b^ |
| ERC2 | 294.813 ± 118.560^c^ | 32.418 ± 5.078^c^ | 294 ± 118^c^ | 1.888 ± 0.710^cd^ | 0.509 ± 0.113^c^ |
| ERC3 | 217.218 ± 70.277^c^ | 32.192 ± 4.032^c^ | 217 ± 70^c^ | 1.298 ± 0.306^d^ | 0.423 ± 0.178^c^ |
| ERC4 | 630.454 ± 232.997^b^ | 54.740 ± 17.037^b^ | 630 ± 232^b^ | 2.663 ± 0.641^bc^ | 0.611 ± 0.073^bc^ |
| ERC5 | 179.866 ± 57.960^c^ | 22.911 ± 11.060^c^ | 179 ± 58^c^ | 1.658 ± 0.154^d^ | 0.494 ± 0.093^c^ |

^#^Groups sharing different letters (a, b, c) indicate significant differences between two treatments by one-way ANOVA test (*P < 0.05*).
